# Hypovitaminosis D Is Prevalent in Patients With Renal AL Amyloidosis and Associated With Renal Outcome

**DOI:** 10.3389/fendo.2022.891712

**Published:** 2022-06-21

**Authors:** Eli Muchtar, Matthew T. Drake, Nelson Leung, Angela Dispenzieri, Martha Q. Lacy, Francis K. Buadi, David Dingli, Suzanne R. Hayman, Prashant Kapoor, Yi Lisa Hwa, Amie Fonder, Miriam Hobbs, Wilson Gonsalves, Taxiarchis V. Kourelis, Rahma Warsame, Stephen Russell, Ronald S. Go, Moritz Binder, Robert A. Kyle, S. Vincent Rajkumar, Shaji K. Kumar, Morie A. Gertz

**Affiliations:** ^1^ Division of Hematology, Mayo Clinic, Rochester, MN, United States; ^2^ Department of Endocrinology and Kogod Center of Aging, Mayo Clinic College of Medicine, Rochester, MN, United States; ^3^ Division of Nephrology and Hypertension, Mayo Clinic, Rochester, MN, United States

**Keywords:** nephrotic syndrome, dialysis, proteinuria, kidney survival, vitamin D

## Abstract

**Introduction:**

Vitamin D deficiency is common, but no data have been reported on vitamin D levels in light chain (AL) amyloidosis.

**Patients and Methods:**

In this exploratory study, stored serum samples from 173 patients with newly diagnosed AL amyloidosis were analyzed for vitamin studies which included 25-hydroxyvitamin D [25(OH)D], 1,25-dihydroxyvitamin D [1,25(OH)_2_D] and vitamin D binding protein (DBP). Measurements were made by liquid chromatography-tandem mass spectrometry. Kidney survival and overall survival (OS) were assessed in association to vitamin D status.

**Results:**

Cardiac and kidney involvement occurred in 69% and 63% of patients, respectively. 25(OH)D deficiency (<20 ng/mL) was seen in 56.6% of the patients and was notably found among patients with heavy proteinuria (96%), hypoalbuminemia (84.3%) and morbidly obese patients (68.3%). Heavy proteinuria (>5 gr/24-h) and vitamin D supplementation were independent predictors of 25(OH)D level on nominal multivariate regression analysis. 1,25(0H)_2_D deficiency was noted in 37.6% of patients and was independently associated with low eGFR and hypoalbuminemia. Progression to ESRD occurred in 23.7% of evaluable patients. Patients who progressed to ESRD had lower serum 25(OH)D and 1,25(OH)_2_D levels compared to those who did not progress to ESRD. On a multivariate analysis, severe 25(OH)D deficiency was an independent predictor of progression to ESRD as was renal stage, while 1,25(OH)_2_D deficiency was not.

**Conclusions:**

Hypovitaminosis D is common in AL amyloidosis, particularly among patients with heavy proteinuria. Severe 25(OH)D deficiency at time of diagnosis predicts progression to ESRD.

## Introduction

Light chain (AL) amyloidosis is a rare B-cell secreting clonal disorder characterized by circulating immunoglobulin light chains with amyloidogenic properties (1). The clonal disease is typically of low burden, with symptoms arising from organ dysfunction caused by light chain-induced amyloid deposition. Organ involvement varies with heart (70-80%) and kidney (60-80%) being the most frequently involved organs (2, 3). Given the systemic nature of the disease and the often-profound impact on organ function, it is common to find various laboratory abnormalities including renal failure, hypoalbuminemia, and coagulation abnormalities, among others. Patients with nephrotic-range proteinuria are specifically prone to hyperlipidemia and hypothyroidism.

Vitamin D deficiency is assessed by measurement of the storage form of vitamin D, namely 25-hydroxyvitamin D [25(OH)D]. The prevalence of vitamin D deficiency, defined as a 25(OH)D level below the optimal range of <20 ng/ml, has been reported to range from 15-50% within the general United States (US) population (4–8). Identified risk factors in the US for vitamin D deficiency include aging, obesity, physical inactivity, darker skin pigmentation, and reduced sun exposure (8). Although 25(OH)D levels assess for vitamin D sufficiency, the active form of vitamin D is 1,25-dihydroxyvitamin D [1,25(OH)_2_D]. 1,25(OH)_2_D reflects 1-alpha hydroxylation of 25(OH)D, a process that occurs primarily within the kidney. Circulating 1,25(OH)_2_D levels are approximately 1000-fold lower than those of 25(OH)D.

Aside from its roles in maintaining serum calcium levels and skeletal homeostasis, vitamin D has been shown to play an important role in regulation of differentiation, proliferation, apoptosis, metastatic potential and angiogenesis in a variety of malignancies (9–11). Low serum 25(OH)D levels have been associated with an increased incidence of several cancers, including colorectal (12, 13) and breast (14, 15), although findings were not consistent in other studies (16, 17). In addition, low serum vitamin D has been associated with shortened survival in several malignancies, including colorectal (18) and breast cancer (19), multiple myeloma (20), chronic lymphocytic leukemia (21) and non-Hodgkin’s lymphoma (22).

No studies have explored vitamin D levels in light chain (AL) amyloidosis. We therefore performed a study to examine vitamin D levels among patients with AL and to explore the impact vitamin D has in this rare disease.

## Methods

In this study patients with biopsy-proven AL amyloidosis diagnosed between January 1, 2000 and September 30, 2013 were included if they had stored serum samples. The stored serum samples were obtained for vitamin D studies, which included 25-hydroxyvitamin D [25(OH)D], 1,25-dihydroxyvitamin D [1,25(OH)_2_D] and vitamin D binding protein (DBP). All vitamin D measurements were made by liquid chromatography-tandem mass spectrometry (LC-MS/MS). Total 25(OH)D and 1,25(OH)_2_D was assessed by the additive sums of the 25(OH)D2 + 25(OH)D3 and 1,25(OH)_2_D2 + 1,25(OH)_2_D3 components, respectively. Samples obtained for vitamin D studies were collected within 90 days of diagnosis (median 20 days) and were stored at -20°C.

Clinical data were extracted from a prospectively maintained database and from additional chart review. Organ involvement was determined based on consensus criteria (23). Renal stage, as previously reported (24), was used to assess risk of progression to end-stage renal disease (ESRD). Briefly, this staging system incorporates eGFR (at 50 mL/min cutpoint) and 24-h proteinuria (at 5 gr/24-h cutpoint) into 3 stages with increasing risk of progression to ESRD with a higher stage. Fluorescence *in-situ* hybridization (FISH) testing of bone marrow plasma cells utilized in our laboratory has been described in detail previously (25). All patients provided written informed consent to have their medical records reviewed according to Mayo Clinic Institutional Review Board (IRB) requirements and Minnesota state law. The Mayo Clinic IRB approved the study.

The χ2 test and Fisher’s exact test were used to compare differences between continuous variables, and the Wilcoxon signed-rank test was used for nonparametric group comparisons. Survival analysis was done using the Kaplan-Meier method. Cox proportional hazards regression models were used to identify variables associated with kidney survival and overall survival (OS). P values less than 0.05 were considered statistically significant. Statistical analysis was performed on JMP software (SAS, Cary, NC).

## Results

### Baseline Characteristics of the Study Cohort

One hundred and seventy-three patients are included in this study. The median age at diagnosis was 60 years. Men comprised 54.3% of the study population. Organ involvement and other baseline characteristics are listed in **Table 1**. Fifty patients (29%) were receiving vitamin D supplementation at the time of vitamin D measurement sampling. The median dose (available for 21 patients) was 1000 IU/day.

**Table 1:** Baseline characteristics of the entire cohort (N=173).

Age, median in years, (range)	60 (32–85)
Male sex, N (%)	94 (54%)
Autologous stem cell transplant as first line treatment	103 (60%)
Involved organs, N (%) Cardiac Renal Hepatic Gastrointestinal Nerve Other >1 organ	119 (69%) 109 (63%) 29 (17%) 27 (16%) 27 (16%) 14 (8%) 106 (61%)
Lambda restricted, N (%)	130 (75%)
Bone marrow plasma cells, % median (IQR)	10 (5–16)
dFLC, mg/L, median (range)	250 (0-20,770)
Serum creatinine, mg/dL, median (IQR)	1.1 (0.9-1.5)
Cardiac stage, % I/II/IIIA/IIIB	19/38/22/21
BMI, kg/m^2^, median (IQR)	26.3 (23.7-29.9)
FISH abnormalities (n=52) t (11;14) Deletion 13q/Monosomy 13 Trisomies IgH translocation without identifiable partner Deletion 17p/Monosomy 17 t (4;14) t (6;14)	53.7% (29/54) 34% (17/50) 22.6% (12/53) 13% (7/54) 0 3.8% (2/53) 3.8% (2/53)

BMI, Body mass index; dFLC, difference between involved and uninvolved light chains; FISH, Fluorescence in-situ hybridization; IQR, Interquartile range.

### Vitamin D Measurements

#### 25(OH)D

The median serum 25(OH)D level was 17 ng/mL (IQR 11-29; normal >20 ng/mL). Vitamin D deficiency, defined as a 25(OH)D level below 20 ng/mL, was found in 56.6% (98/173) of the study population. Severe vitamin D deficiency (25(OH)D <10 ng/mL) was noted in 22.5% (39/173) of patients. Serum 25(OH)D was significantly lower in samples collected from November to April (winter months) as compared to samples collected from May to October (summer months; median 13.5 *vs* 19.5 ng/mL; P=0.009). Serum 25(OH)D levels <20 ng/ml were more frequent in individuals with proteinuria >5 grams/24-h (96.1%), serum albumin <2.5 g/dL (84.3%) and body mass index (BMI) >30 kg/m^2^ (68.3%) (**Table 2**). In contrast, vitamin D supplementation at the time of sampling was associated with a lower rate of vitamin D deficiency (38%).

**Table 2:** Prevalence, univariate, and multivariate nominal regression analysis of factors associated with low serum 25 (OH)D.

	% with 25 (OH)D <20 ng/mL	Univariate analysis		Multivariate analysis	
		Odds ratio (95% CI)	P-value	Odds ratio (95% CI)	P-value
Age ≥65 years	63.6%	1.5 (0.8-2.9)	0.2	–	
Male gender	49%	0.6 (0.3-1.1)	0.1	–	
BMI ≥30 kg/m^2^	68.3%	1.9 (0.9-4.0)	0.08	2.3 (0.9-6.0)	0.09
Vitamin D supplementation usage	38%	0.3 (0.2-0.7)	**0.001**	0.3 (0.1-0.7)	**0.005**
Winter month collection	64.1%	1.7 (0.94-3.2)	0.07	1.3 (0.6-3.0)	**0.55**
Heart involvement	52.9%	0.6 (0.3-1.2)	0.14	–	
Liver involvement	48.3%	0.7 (0.3-1.5)	0.32	–	
Nerve involvement	40.7%	0.5 (0.2-1.1)	0.07	0.7 (0.3-2.1)	0.58
Gastrointestinal involvement	66.7%	1.7 (0.7-3.9)	0.24	–	
Proteinuria >5 gr/24-h	96.1%	37.0 (8.6-160.0)	**<0.001**	30.1 (5.7-159.7)	**<0.001**
eGFR <30 ml/min/1.73 m^2^	60%	1.2 (0.5-3.0)	0.74	–	
Serum albumin <2.5 g/dL	84.3%	8.3 (3.5-19.4)	**<0.001**	1.8 (0.5-5.9)	0.34
Lambda restricted disease	60.8%	2.0 (0.97-3.9)	0.058	0.4 (0.2-1.2)	0.1
dFLC ≥ 180 mg/L	46.8%	0.8 (0.4-1.5)	0.44	–	

BMI, Body mass index; CI, Confidence interval; dFLC, difference between involved to uninvolved light chains; eGFR, estimated glomerular filtration rate. Bold indicate statistical significance at <0.05.

#### 1,25(OH)_2_D

The median serum level of 1,25(OH)_2_D was 23 pg/mL (IQR 12-32; normal 18-64 pg/mL for men; 18-78 pg/mL for women). 1,25(OH)_2_D levels below the normal range (18 pg/mL) were found in 37.6% (65/173) of patients. The highest rate of 1,25(OH)_2_D deficiency (<18 pg/mL) was found among individuals with eGFR <30 ml/min/1.73m^2^ (85%, **Table 3**). 1,25(OH)_2_D levels did not differ based on the season of sample collection (median 20.3 *vs* 24 ng/mL; P=0.21). There was a weak correlation between 25(OH)D and 1,25(OH)_2_D levels (r=0.18, P=0.01). Overall, 42.3% of patients had both 25(OH)D and 1,25(OH)_2_D below normal range.

**Table 3:** Prevalence, univariate, and multivariate nominal regression analysis of factors associated with low serum 1,25 (OH)_2_D levels.

	% with 1,25 (OH)_2_D <18 pg/mL	Univariate analysis		Multivariate analysis	
		Odds ratio (95% CI)	P-value	Odds ratio (95% CI)	P-value
Age ≥65 years	40.4%	1.6 (0.8-3.1)	0.15	–	
Male gender	45.5%	1.3 (0.7-2.4)	0.43	–	
BMI ≥30 kg/m^2^	53.7%	2.4 (1.2-4.8)	**0.01**	2.0 (0.8-4.8)	0.12
Vitamin D supplementation usage	28%	0.5 (0.3-1.1)	0.09	0.6 (0.3-1.5)	0.26
Winter month collection	40.3%	1.2 (0.7-2.2)	0.54		
Heart involvement	41.5%	1.7 (0.8-3.4)	0.13	–	
Liver involvement	41.3%	1.2 (0.5-2.7)	0.66	–	
Nerve involvement	37%	1.0 (0.4-2.3)	0.92	–	
GI involvement	37%	1.0 (0.4-2.3)	0.92	–	
Proteinuria >5 gr/24-h	64%	4.8 (2.4-9.7)	**<0.001**	1.9 (0.6-5.7)	0.25
eGFR <30 ml/min/1.73 m^2^	85%	12.3 (3.4-43.9)	**<0.001**	8.7 (2.2-34.3)	**0.002**
Serum albumin <2.5 g/dL	64.7%	5.2 (2.5-10.6)	**<0.001**	3.8 (1.3-10.9)	**0.01**
Lambda restricted disease	39.5%	1.4 (0.7-2.8)	0.41	–	
dFLC ≥ 180 mg/L	41%	1.3 (0.6-2.5)	0.53	–	

BMI, Body mass index; CI, Confidence interval; dFLC, difference between involved to uninvolved light chains; eGFR, estimated glomerular filtration rate. Bold indicate statistical significance at <0.05.

### Vitamin D Binding Protein (DBP)

DBP measurements were available for 87 patients (50.3% of study population). The median level of serum DBP was 57 μg/mL (IQR 35 – 120 μg/mL), significantly lower than the normal range (168-367 μg/mL). Low levels of 25(OH)D or 1,25(OH)_2_D were not associated with lower DBP levels. Patients with proteinuria >5 gr/24-h had similar levels of serum DBP compared to patients with ≤5 gr proteinuria/24-h (median 73 *vs* 50 μg/mL; P=0.11). Median levels were below normal in both groups. In comparison, patients with proteinuria >5g/24-h had lower serum albumin levels than those with proteinuria ≤5g/24-h (median 1.9 *vs* 3.2 g/dL; P<0.001). Females had borderline significantly lower DBP levels than men (median 48 *vs* 72 μg/mL; P=0.06). No other factors were found to correlate with DBP levels.

### Host and Disease Factors Associated With Vitamin D Deficiency

**Figure 1:**
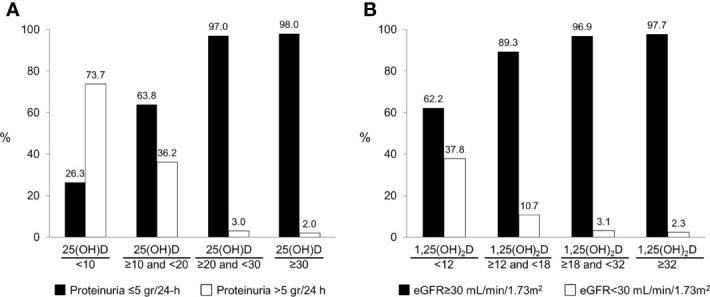
The association between vitamin D measurements and renal parameters: **(A)** The distribution of 25(OH)D groups based on level of proteinuria. **(B)** The distribution of 1,25(OH)_2_D groups based on level of estimated glomerular filtration rate.

25(OH)D levels <20 ng/mL were strongly associated with vitamin D supplementation usage at the time of sampling (OR 0.3, 95% CI 0.2-0.7; P=0.001), proteinuria >5 grams/24-h (OR 37.0, 95% CI 8.6-160.0; P<0.001) and serum albumin <2.5 g/dL (OR 8.3, 95% CI 3.5-19.4; P<0.001) (**Table 2**). On multivariate analysis, vitamin D usage at sampling (OR 0.3, 95% CI 0.1-0.7, P=0.005) and proteinuria >5 grams/24-h (OR 30.1, 95% CI 5.7-159.7; P<0.001) remained independent predictors for 25(OH)D levels <20 ng/mL. **Figure 1A** depicts the relation between 25(OH) D level groups (<10, 10-19.9, 20-29.9, ≥30 ng/mL) and patients with or without significant proteinuria (cutoff at >5 gram/24-h), demonstrating the correlation between heavy proteinuria and lower levels of 25(OH)D.

Four factors were associated with an increased likelihood of 1,25(OH)_2_D levels below the lower limit of normal (18 pg/mL) on univariate analysis (**Table 3**). These included: BMI ≥30 kg/m^2^ (OR 2.4, 95% CI 1.2-4.8; P=0.01), proteinuria >5 grams/24-h (OR 4.8, 95% CI 2.4-9.7; P<0.001), eGFR <30 ml/min/1.73 m^2^ (OR 12.3, 95% CI 3.4-43.9; P<0.001) and serum albumin <2.5 g/dL (OR 5.2, 95% CI 2.5-10.6; P<0.001). On multivariate analysis, eGFR <30 ml/min/1.73 m^2^ (OR 8.7, 95% CI 2.2-34.3; P=0.002) and serum albumin <2.5 g/dL (OR 3.8, 95% CI 1.3-10.9; P=0.01) retained independent associations with low 1,25(OH)_2_D levels. **Figure 1B** demonstrates the strong correlation between 1,25(OH) D level groups and eGFR.

### FISH Findings and Vitamin D Measurements

FISH testing was available for 55 patients (31.8% of study population). The most common FISH abnormalities were t(11,14) found in 53.7% of patients followed by chromosome 13 abnormalities (34%) and trisomies in 22.6% of patients. Patients with t(11,14) were more likely to have 25(OH)D levels ≥20 ng/mL than patients with non-t(11,14) (65.5% *vs* 40%), but this reached only borderline statistical significance (P=0.059). Similarly, 1,25(OH)_2_D levels ≥18 pg/mL were also associated with t(11,14) status (75.9% for t(11,14)-positive patients versus 52% in non-t(11,14) patients; P=0.06). No other association was found between vitamin D serum measurements and FISH findings.

### The Association of Vitamin D Measurements With Kidney Survival

Data on kidney survival [progression to end-stage renal disease (ESRD)] was ascertained for 162 patients (94% of the study population). Ten patients presented or progressed to ESRD within 3 months of AL diagnosis and were excluded from this analysis. Of the remaining patients, 36 patients developed ESRD, representing 23.7% of patients assessed for progression to ESRD. Patients who developed ESRD had lower baseline 25(OH)D levels compared to patients who did not progress to ESRD (median 10 *vs* 21 ng/mL; P<0.001). Similarly, baseline 1,25(OH)_2_D was lower in patients who developed ESRD compared to those who did not (median 15.9 *vs* 26.4 pg/mL; P<0.001).

Univariate and multivariate Cox proportional regression analyses assessing the risk of progression to ESRD and incorporating renal stage and vitamin D measurements (selected close to median values for the ESRD group) are provided in **Table 4**. 25(OH)D <10 ng/mL was an independent predictor for ESRD alongside renal stage, while 1.25 (OH)_2_D <18 pg/mL was not.

**Table 4:** Univariate and multivariate Cox nominal logistic regression analyses for predictors of progression to end-stage renal disease.

	Univariate analysis		Multivariate analysis	
	Odds ratio (95% confidence interval)	P-value	Odds ratio (95% confidence interval)	P-value
Renal stage I II III	Reference 4.9 (2.0-12.3) 62.8 (18.9-208.8)	**<0.001** **<0.001**	Reference 3.6 (1.4-9.3) 39.1 (10.5-145.2)	**0.008** **<0.001**
25 (OH)D <10 ng/mL	4.1 (2.1-7.8)	**<0.001**	2.1 (1.03-4.4)	**0.04**
1,25 (OH)_2_D <18 pg/mL	4.0 (2.1-7.8)	**<0.001**	1.5 (0.7-3.3)	0.26

Bold indicate statistical significance at <0.05.

### The Association of Vitamin D Measurements With Overall Survival

**Figure 2:**
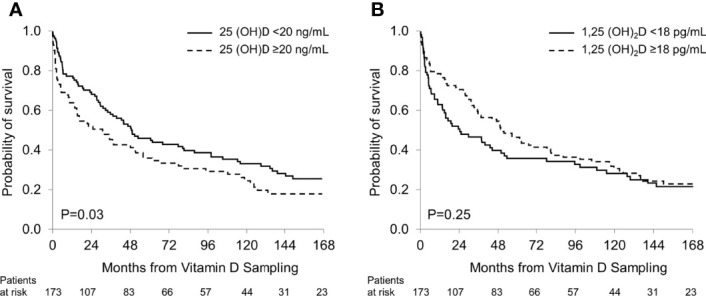
Overall survival based on: **(A)** 25(OH)D groups; **(B)** 1,25(OH)_2_D groups.

Over the study period 140 patients died, representing 81% of the study cohort. The median follow-up was 16.3 years. No patient was lost to follow-up. Patients with 25(OH)D levels >20 ng/mL had shorter overall survival (OS) compared to patients with lower 25(OH)D values (median 31.1 *vs* 49.2 months; P=0.03; **Figure 2A**), reflecting a strong correlation between low 25(OH)D levels and a favorable prognosis in patients with renal AL amyloidosis. There was no significant difference in OS between patients with 1,25 (OH)_2_D ≥18 pg/mL and those with lower levels (median 51.0 *vs* 24.2 months; P=0.25; **Figure 2B**).

In multivariate analyses for OS, a 25(OH)D level <20 ng/mL showed a trend towards longer OS (OR 0.7, P=0.07), whereas a 1,25(OH)_2_D level <18 pg/mL did not (**Table 5**).

**Table 5:** Univariate and multivariate Cox proportional regression analyses for predictors for overall survival.

	Univariate analysis		Multivariate analysis	
	Odds ratio (95% confidence interval)	P-value	Odds ratio (95% confidence ikidenynterval)	P-value
Age >65	1.8 (1.2-2.5)	**0.001**	1.5 (1.04-2.3)	**0.03**
Cardiac involvement	2.5 (1.7-3.6)	**<0.001**	2.2 (1.5-3.2)	**<0.001**
Autologous stem cell transplantation as initial therapy	0.4 (0.3-0.5)	**<0.001**	0.5 (0.3-0.7)	**0.002**
25 (OH)D <20 ng/mL	0.7 (0.5-0.97)	**0.03**	0.7 (0.5-1.04)	0.08
1,25 (OH)_2_D <18 pg/mL	1.3 (0.9-1.8)	0.12	1.1 (0.7-1.6)	0.67

Bold indicate statistical significance at <0.05.

## Discussion

In this study we explored, for the first time, the prevalence and risk factors for vitamin D deficiency in patients with AL amyloidosis. We found that more than half of our study population had vitamin D deficiency (defined as 25(OH)D <20 ng/mL), while over a fifth of the study cohort had severe vitamin D deficiency [25(OH)D <10 ng/mL]. Vitamin D deficiency was particularly common among patients with significant proteinuria, an established risk factor for vitamin D deficiency. We have also found that severe vitamin D deficiency was associated with increased risk for progression to end-stage renal disease. Overall, the findings in this study support measurement of baseline vitamin 25(OH)D in patients with AL amyloidosis, particularly among those with significant proteinuria.

The proportion of patients with vitamin D deficiency in this study (56.6%) is higher compared to the general US population. In the National Health and Nutrition Examination Survey (NHANES), conducted in a similar time period to our study (2001 to 2010), the prevalence of vitamin D deficiency among US adults age 18 or above ranged between 27.1% and 30.8% (depending on age group) (8). Although our sample size was small, it was powered to identify seasonal variations in 25(OH)D levels and a positive effect of oral vitamin D supplementation on serum level. Such a high prevalence of vitamin D deficiency has been found in general medical inpatients (26) and populations with poor health (27) and corresponds to the poor and complex health issues that patients with AL amyloidosis often encounter.

We identified several risk factors for hypovitaminosis D in AL amyloidosis. The most significant risk factor for low 25(OH)D levels was significant proteinuria. Patients with nephrotic syndrome are known to be at risk for vitamin D deficiency (7). Kidney involvement is a common disease manifestation in AL amyloidosis with nephrotic range proteinuria present in approximately 30% of patients at time of diagnosis (28), explaining in part the high prevalence of vitamin D deficiency in this disease. Urinary loss of vitamin D bound to its carrier protein (DBP) and to albumin in patients with nephrotic syndrome has been implicated as a main cause for vitamin D deficiency in patients with nephrosis (29). In this study, serum level of DBP was below the lower limit of normal, supporting this hypothesis. However, serum DBP levels were not lower in patients with significant proteinuria when compared to those who without. Since serum albumin was significantly lower in those with significant proteinuria, it may be that urinary loss of albumin with vitamin D bound to it is the main mechanism to explain hypovitaminosis D. The reason for low DBP in almost all AL patients requires further investigation but given that it has a molecular weight of 52-59 kDa, which is slightly lower than that of albumin (66.5 kDa), it is likely that increased urinary losses are a major contributing factor. In addition, other factors may alter vitamin D levels in patients with AL amyloidosis, including poor GI absorption due to intestinal edema, impairment in hepatic 25-hydroxylation and reduced sun exposure related to poor health.

In this study, we have shown that severe 25(OH)D deficiency is an independent predictor of progression to ESRD. This finding has been reported in previous studies with patients with chronic kidney disease (30–34). The mechanism by which vitamin D deficiency leads to loss of renal function remains elusive. However, it has been postulated that vitamin D may be renoprotective *via* modulation of several pathways including the renin-angiotensin, NF-kB and Wnt/β-catenin signaling pathways (35). In addition, vitamin D deficiency can downregulate the generation of nephrin and podocin, essential structures of the podocytes, an effect which would then be expected to damage the glomerular filtration barrier leading to a pro-proteinuric effect (36). However, as low vitamin D levels were associated with higher renal stage, one cannot rule out that vitamin D deficiency is not directly involved in progression to ESRD, but rather represents a marker of advanced renal impairment and progression to ESRD.

Irrespective of the mechanisms by which vitamin D deficiency increases the risk of ESRD, correction of vitamin D deficiency in patients with CKD (including renal AL amyloidosis), should be assessed in prospective studies given the potential benefit of such treatment. A large, randomized trial among type II diabetes mellitus participants did not demonstrate a renoprotective effect for vitamin D supplementation versus placebo (37). However, in that study participants had a high baseline eGFR and less than 20% of the participants had vitamin D deficiency at baseline. A small retrospective study among kidney transplant participants also failed to demonstrate kidney allograft protection with vitamin D supplementation (38).

25(OH)D deficiency, but not 1,25(OH)_2_D deficiency, was associated with improved survival compared to patients with normal vitamin D levels. This observation stands in contrast to observations among cancer patients (12–15, 18–22). This is likely a reflection of association between 25(OH)D deficiency and renal involvement, specifically with a higher degree of proteinuria. In AL amyloidosis, patients with single-organ renal involvement have higher degree of proteinuria compared with AL patients with multi-organ involvement (28). As survival in AL amyloidosis is driven primarily by cardiac involvement (2, 39, 40) and number of involved organs (28), vitamin D 25(OH) deficiency likely serves as a confounder for a more prognostically favorable patient profile.

In this study there was a trend for 25(OH)D and 1,25(OH)_2_D levels to be normal in subjects with t(11:14) as compared to those with non-t(11,14). t(11,14) is the most common genetic abnormality in patients with AL amyloidosis (25). Although it may be a coincidental finding without an underlying mechanism, it is possible that this finding may reflect the effect that active vitamin D derivatives, mainly 1,25(OH)_2_D have on cell apoptosis (41–43). As a genetic aberration, t(11,14) confers an anti-apoptotic effect by inducing, *via* an unknown mechanism, the upregulation of B-cell lymphoma-2 (BCL-2) (44), an anti-apoptotic protein. Therefore, one hypothesis that may be explored is whether vitamin D deficiency has an anti-apoptotic effect that leads to the selection of plasma cell clones which are less apoptosis resistant. Our study was not designed to answer this question.

Our study has several strengths. These include that this is the first report on the incidence of vitamin D deficiency in patients with AL amyloidosis, and the ability to measure vitamin D metabolites and vitamin D binding protein *via* the gold standard method of liquid chromatography-tandem mass spectrometry. We were also powered for several observations including the positive effect of concurrent vitamin D supplementation on serum 25(OH)D and the expected seasonal variation on serum 25(OH)D levels. The study follow-up period was long (median >16 years), allowing sufficient time of observation for events (ESRD, death). The primary limitation to our study was its retrospective design. We did not preselect number of samples based on predefined hypothesis, which may limit the study power. These limitations negatively affect the generalizability of the above findings and require further studies.

In conclusion, we have shown that patients with renal AL amyloidosis have a high incidence of vitamin D deficiency. We anticipate our findings will serve as the basis for future prospective clinical efforts to improve vitamin D levels in these patients.

## Data Availability Statement

The raw data supporting the conclusions of this article will be made available by the authors, without undue reservation.

## Ethics Statement

The studies involving human participants were reviewed and approved by Mayo Clinic Institutional review Board. The patients/participants provided their written informed consent to participate in this study.

## Author Contributions

EM, MTD, and MG designed the study, analyzed the data, wrote the first draft and approved the final version of the manuscript; NL, AD, ML, FB, DD, SH, PK, YH, AF, MH, WG, TK, RW, SR, RG, MB, SR, and SK provided care for patients, revised the manuscript critically, and approved the final version of the manuscript. RK performed patients’ follow-up, revised the manuscript critically, and approved final version of the manuscript. All authors contributed to the article and approved the submitted version.

## Funding

This work was supported in part by CA90628-21 Pal Calabresi K12 Career Development Award.

## Conflict of Interest

EM: honorarium from Janssen and consultation fee from Protego (fee paid to institution). NL served on advisory board for Takeda Pharmaceuticals. AD: research funding from Celgene, Millennium Pharmaceuticals, Pfizer, and Janssen and received a travel grant from Pfizer. ML: Research Funding from Celgene. PK is a principal investigator of research studies for which Mayo Clinic has received funding from AbbVie, Takeda, Sanofi, Janssen, Karyopharm, Glaxo SmithKline, Regeneron Pharmaceuticals, Ichnos Sciences and Amgen. He has served on the Medical advisory board meetings of Sanofi, Pharmacyclics, BeiGene, Cellectar, GSK, X4 and Karyopharm. SK received research funding for clinical trials to the institution from Abbvie, Amgen, BMS, Carsgen, Janssen, Astra-Zeneca, Novartis, Roche-Genentech, Takeda, Tenebio, Molecular Templates, received consulting/Advisory Board participation (with no personal payments) from Abbvie, Amgen, BMS, Janssen, Roche-Genentech, Takeda, Astra-Zeneca, Bluebird Bio, Epizyme, Secure Biotherapeutics and (with personal payment) Oncopeptides, Beigene, Antengene. MG served as a consultant for Millennium Pharmaceuticals and received honoraria from Celgene, Millennium Pharmaceuticals, Onyx Pharmaceuticals, Novartis, GlaxoSmithKline, Prothena, Ionis Pharmaceuticals, and Amgen.

The remaining authors declare that the research was conducted in the absence of any commercial or financial relationships that could be construed as a potential conflict of interest.

## Publisher’s Note

All claims expressed in this article are solely those of the authors and do not necessarily represent those of their affiliated organizations, or those of the publisher, the editors and the reviewers. Any product that may be evaluated in this article, or claim that may be made by its manufacturer, is not guaranteed or endorsed by the publisher.
